# eIF5A inhibition influences T cell dynamics in the pancreatic microenvironment of the humanized mouse model of Type 1 Diabetes

**DOI:** 10.1038/s41598-018-38341-5

**Published:** 2019-02-07

**Authors:** Shahnawaz Imam, R. Prathibha, Pervaiz Dar, Khalil Almotah, Ahmed Al-Khudhair, Syed Abdul-Moiz Hasan, Nancy Salim, Talha Naser Jilani, Raghavendra G. Mirmira, Juan Carlos Jaume

**Affiliations:** 1 Division of Endocrinology, Diabetes and Metabolism, Department of Medicine, College of Medicine and Life Sciences, University of Toledo, Toledo, OH USA; 2 Center for Diabetes and Endocrine Research (CeDER), Department of Medicine, College of Medicine and Life Sciences, University of Toledo, Toledo, OH USA; 3 Department of Pediatrics, Indiana University School of Medicine, Indianapolis, IN USA; 4 Faculty of Veterinary Sciences and Animal Husbandry, Sher-e-Kashmir University of Agricultural Sciences and Technology of Kashmir (SKUAST-K), Shuhama, Srinagar, 190006 Jammu and Kashmir India

## Abstract

We have developed a transgenic mouse model of Type 1 Diabetes (T1D) in which human GAD65 is expressed in pancreatic β-cells, and human MHC-II is expressed on antigen presenting cells. Induced GAD65 antigen presentation activates T-cells, which initiates the downstream events leading to diabetes. In our humanized mice, we have shown downregulation of eukaryotic translation initiation factor 5 A (elF5A), expressed only in actively dividing mammalian cells. *In-vivo* inhibition of elF5A hypusination by deoxyhypusine synthase (DHS) inhibitor “GC7” was studied; DHS inhibitor alters the pathophysiology in our mouse model by catalyzing the crucial hypusination and the rate-limiting step of elF5A activation. In our mouse model, we have shown that inhibition of eIF5A resets the pro-inflammatory bias in the pancreatic microenvironment. There was: (a) reduction of Th1/Th17 response, (b) an increase in Treg numbers, (c) debase in IL17 and IL21 cytokines levels in serum, (d) lowering of anti-GAD65 antibodies, and (e) ablation of the ER stress that improved functionality of the β-cells, but minimal effect on the cytotoxic CD8 T-cell (CTL) mediated response. Conclusively, immune modulation, in the case of T1D, may help to manipulate inflammatory responses, decreasing disease severity, and may help manage T1D in early stages of disease. Our study also demonstrates that without manipulating the CTLs mediated response extensively, it is difficult to treat T1D.

## Introduction

The hallmark of type 1 diabetes (T1D) is immune-mediated destruction of insulin secreting β-cells of the pancreatic islets of Langerhans, resulting in hyperglycemia and lifelong dependency on exogenous insulin. T1D develops in individuals having familial genetic susceptibility under certain intrinsic and/or environmental influences that are not fully understood. Immunological events, although not precisely defined, are thought to involve innate immune activation and adaptive T and B cell responses against various β-cell antigens^1^. T cells have been well recognized as key orchestrators of T1D in mouse models as well as in human patients. T cell dynamics in the islet microenvironment is characterized by T helper (Th) 1 and Th17 cell bias and/or a T-regulatory cell (Treg) defect that ultimately culminates into CTL mediated destruction of the β-cells^2–6^.

Recent studies recognize the role of Th17 cells in the mediation of T1D; coupling this information with earlier studies^7,8^ implies the dominant, yet not causal, the role of Interferon (IFN) γ and Th1 cells with the mediation of T1D in neonatal NOD mice^9,10^. Further studies indicate when IFNγ is blocked with a neutralizing antibody at an early stage, the disease is exacerbated^11^. Th17 cells are reported to be elevated in the peripheral blood and pancreatic lymph nodes of T1D patients as compared to healthy humans^3,12,13^. Both Th1 and Th17 cells seem to cooperate in the mediation of T1D. Th1 cells or IFNγ is often associated with an increased expression of Th17 cells^14^. IL17/IFNγ receptor double-deficient mice show significantly delayed the onset of diabetes compared to IL17 single knockout mice^15^. Another key player in the pro-inflammatory/anti-inflammatory dyad of immunity is the Tregs. Pancreatic Tregs in mice have been shown to be affected at both the numerical and functional levels in diabetic NOD mice^16^. Tregs in peripheral blood of human patients display increased sensitivity to apoptosis and are functionally defective^17–21^. Notably, T helper subsets are now considered more plastic than previously appreciated and have demonstrated great flexibility in their differentiation options^22–24^. In adoptive transfer models, islet antigen-specific Th17 cells have been shown to convert into Th1-like cells to induce diabetes^23,25^. Marwaha *et al*. (2010) reported that Treg cells acquire a Th17 like phenotype in human T1D patients^12^. However, it is not yet clear if this reprogramming is required for T1D induction or if it is instead a consequence of the immune/inflammatory response.

Nevertheless, T helper cells cannot directly interact with the β-cells due to their lack of MHC class II expression and are unable to cause direct cytotoxicity. They contribute to β-cell death through soluble mediators like cytokine and other death signals (eg IL1, TNFa, IFNγ, FASL). These inflammatory mediators initiate multiple pathways in β-cells, including endoplasmic reticulum (ER) stress, leading to their dysfunction and eventual death through apoptosis^26–28^. It is still debatable whether ER stress is a cause or consequence of β-cell dysfunction in T1D.

Currently, CTLs are considered prime contributors of β-cell death in T1D which is supported by numerous studies involving both mice and humans^6,29–31^. In mouse models, efficient adoptive transfer of T1D typically requires cluster of differentiation of (CD) 8^+^ T cells^32,33^, and diabetes is prevented in MHC class I or β 2-microglobulin deficient mice^34^. In humans, β-cell antigen-specific CD8 T cell expansions have been identified in the peripheral blood of T1D patients^35,36^ and CD8 T cells predominate the cellular infiltrate of the spleen from cadaveric T1D donors^37,38^.

In the present study, we tested the effect of inhibiting eukaryotic translation initiation factor 5 A (eIF5A) by *N*1-guanyl-1,7-diaminoheptane (GC7) on the T cell dynamics in the pancreas and local lymph nodes of our humanized mouse model of T1D. GC7 targets the deoxyhypusine synthase (DHS) enzyme which catalyzes hypusination of eIF5A, which is mandatory for its activity and functions^39^. Few studies have indicated the role of hypusinated eIF5A in adaptive immunity through its effect on T- and B-cell proliferation^40,41^, and maturation of DCs^42^. Hypusinated eIF5A has been shown to promote cytokine-mediated β-cell dysfunction through post-transcriptional regulation of iNOS^43^. Earlier studies by our group have indicated that eIF5A inhibition delays the onset of T1D in our humanized mouse model^44^ as well as in NOD mice^45^. Here, we show that eIF5A inhibition alters T cell profiles, especially T helper subsets, in the pancreas and draining lymph nodes of the mouse model. We also show that eIF5A inhibition reduces the ER stress in the pancreatic microenvironment and improves the functionality of β-cells.

## Material and Methods

### Experimental Design

Two groups of our transgenic humanized mice of T1D (*n* = 6–8 per group) were used comprising of an equal number of males and females^46,47^. The diabetes was confirmed by hyperglycemia (>250 mg/dl for two consecutive days) and a positive glucose tolerance test (GTT). After the confirmation of diabetes, animals in one group were treated with GC7 for elF5A inhibition through intra-peritoneal route from 14 weeks through 22 weeks of age at the dose rate of 4 mg/kg body weight for 5 days a week and served as the treatment group. Animals in the other group were given placebo (normal saline) in a similar manner and served as a nontreated group. The animals in both the groups and their glucose levels were monitored weekly during the whole experiment. All the animals in both the groups were euthanized at 28 weeks of age to collect the spleen (SP), inguinal lymph node (IGLN), peri-pancreatic lymph node (PPLN) and pancreas (PN) (Sketch 1).

### Mice

Humanized mouse model of T1D consisting of Murine MHC-class II deficient (mII^_^), HLA-DQA1*0301/DQB1*0302 (DQ8), and hGAD65 transgenic mice^48^ in BTBR background^49^ were used in this study^46,47^. DQ8 and hGAD65 homozygosity was determined as previously described^44,48–50^. The Institute Animals Care and Use Committee (IACUC), approved all animal protocols. All procedures were performed in accordance with the relevant guidelines and regulations from the University of Toledo.

### Glucose tolerance test (GTT) and glucose-stimulated insulin secretion (GSIS)

For GTT, animals fasted for eight hours and then given an intraperitoneal injection of 2 g glucose per kg body weight. Blood glucose was measured by Ascensia Breeze Glucometer (Bayer) at 0, 20, 30, 60, 90, 120, 150, 180 and 210 min. intervals via tail vein puncture method. In our experiment, the GTT was performed at two-time points; at the age of 12–14 weeks (pre GC7 administration) and another at the age of 25–26 weeks (post GC7 administration). Following the glucose challenge, we also measured plasma insulin concentrations at 0, 10 and 30 min. using a mouse ultrasensitive insulin ELISA kit (Crystal Chem, Inc., USA).

### Pancreatic insulin content

Insulin was extracted from the pancreas (PN) by a standard acid-ethanol extraction method as described earlier^51^. Insulin concentration in the extract was determined using a Mouse Ultrasensitive Insulin ELISA kit (Crystal Chem, Inc., USA). The insulin content was standardized to the total protein concentration measured by Qubit Protein Assay kit (Invitrogen).

### Enzyme-linked immunosorbent assays

We quantified anti-GAD65 antibodies and cytokine (IL17 and IL21) in the serum of the animals. Anti-GAD65 antibodies were measured with GAD65 ELISA kit (Kronus, Star, ID) according to the manufacturer’s instructions. IL17 and IL21 concentrations were measured using Mouse IL17 and IL21 Platinum ELISA kit (eBioscience, San Diego, CA). At least two independent experiments in duplicated wells were performed, and average values for each sample were obtained for data analysis.

### Flow cytometry

The tissues were mechanically homogenized to form single cell suspensions. Cell surface staining was performed by incubating 5 × 10^6^ cells with fluorochrome-conjugated antibodies against mouse CD3 (clone 145-2C11, APC, APCCy7), CD4 (clone H129.19, PECy5), CD8 (clone 53-6.7, PECy7), CD25 (clone PC61, PE), (BD Biosciences, San Jose, CA) or isotype controls for 20 min. on ice. The cells were washed and fixed in 1% paraformaldehyde. A subset of cells were permeabilized with cytofix/cytosperm fixation and permeabilization solution (BD Biosciences). Intracellular staining was performed with fluorochrome-conjugated antibodies against mouse IL17 (clone 559502, PE), IFNγ (clone 554413, APC) and forkhead box P3 (FOXP3) (clone MF23, Alexa Fluor 488, Alexa Fluor 647) as previously described^44^. Hoechst 33342 (10 µg/ml) staining was performed to gate live cells containing 2n-4n cellular DNA. Stained cells were acquired by BD LSR II /BD FACSAria II (BD Biosciences) flow cytometers. The data were analyzed using FlowJo software (Tree Star Inc.)^44^.

### Quantitative RT-PCR analysis

Total RNA was extracted from the frozen pancreatic tissue using TRIzol (Invitrogen) as per manufactures instructions. One µg of RNA was converted into cDNA using iScript Reverse Transcription kit. eIF5A and ER stress-related gene expression was quantified using SYBR Green chemistry (Applied BioSystems, Life technologies) with specific primers (Table 1). The expression was quantified using the ΔΔCT method with 18 S RNA/*GAPDH* as the endogenous control. Minus-reverse transcriptase samples were used as negative controls to test for DNA contamination.

**Table 1:** Quantitative real time PCR primers for ER stress genes.

Mouse *BiP/Hspa5*_ex4-5_F	GGCGTATTTGGGAAAGAAGGT
Mouse *BiP/Hspa5*_ex4-5_R	GCATCATTGAAGTAAGCTGGTACAG
Mouse *Wfs1*_ex6-7_F	CGCCTCGTCAGCAGTGAAT
Mouse *Wfs1*_ex6-7_R	GGAACAGGTTGGTGGGAATG
Mouse *Ero1l*_ex2-3_F	AACTACAGACTTTTCCCAAGACTACAAA
Mouse *Ero1l*_ex2-3_R	GGTTGATGTCATTCCAGAAAGGA
Mouse *CHOP/Ddit3*_ex3-4_F	GGTCCTGTCCTCAGATGAAATTG
Mouse *CHOP/Ddit3*_ex3-4_R	TAGGGACGCAGGGTCAAGAG
Mouse total *XBP-1*_F	TGGCCGGGTCTGCTGAGTCCG
Mouse total *XBP-1*_R	GTCCATGGGAAGATGTTCTGG;
Mouse spliced *XBP-1*_F	CTGAGTCCGAATCAGGTGCAG
(original CAG was mutated to AAT to reduce background signal from unspliced *XBP-1*)	
Mouse spliced *XBP-1*_R	GTCCATGGGAAGATGTTCTGG
Mouse 18S_RNA_F	CGCCGCTAGAGGTGAAATTCT
Mouse 18S_RNA_R	CGAACCTCCGACTTTCGTTC
Mouse eIF5A_ F1	CCCAACATCAAACGGAATGAC (8)
Mouse eIF5A_ R1	GCAGACGAAGGTCCTCTCGTA (8)

### Hematoxylin/Eosin (H&E) staining of pancreases

After sacrifice, a part of the pancreas was embedded into Tissue-Plus O.C.T. Compound (Fisher Scientific Co. L.L.C, Pittsburgh, PA) for morphological analysis of pancreatic islets and insulitis degree classification. Treated and nontreated pancreas samples were sectioned at 10 um. The homogenous pancreatic sections were stained with hematoxylin/eosin (H&E) and slides were analyzed by optical microscope for histological identification, localization of lymphocytic infiltration and for classification of the islets with disturbed architecture^50^.

### Western blot

#### Western blot of the protein expressed by CD4 and CD8 lymphocytes

Untouched CD4 and CD8 T cells were isolated from single cell suspensions of mouse peri pancreatic lymph nodes (LN) and spleen (S) using CD4/CD8 T cell isolation kit (Miltenyi Biotech, USA). The enriched single positive CD4 or CD8 cells were lysed and protein concentration was determined using commercially available BCA protein estimation kit (Pierce Biotechnology). Lysate protein (25–50 ug) was resolved over 8% to 14% Tris-glycine polyacrylamide gel and then transferred onto the nitrocellulose membrane. The blot was blocked with 5% nonfat dry milk/3% bovine serum albumin (BSA) and further incubated with primary antibodies against mice DHPS (Cat#ab202133, Abcam, Cambridge, United Kingdom), Hypusinated elF5A (Cat#ABS1064, Millipore Sigma, Burlington, MA), elF5A (Cat#ab137561, Abcam, Cambridge, United Kingdom) and α/β-Tubulin (Cat#2148 S, Cell Signaling, Danvers, MA). The blots were washed with washing buffer and further incubated with horseradish peroxidase-labeled secondary antibodies (Amersham Life Sciences, Little Chalfont, United Kingdom). The intensities of the blots were detected by chemiluminescence using an enhanced chemiluminescence kit (Amersham Life Science) followed by autoradiography. For every immunoblot, equal concentration and loading volume were confirmed by stripping the blot and re-incubation with Tubulin antibody. The densities of the proteins were quantified by using Image J software (NIH, USA). The density of expressed proteins was normalized by dividing the density of tubulin of the same sample. The data were statistically analyzed in the SAS 9.3 software.

### Statistical analysis

Data for glucose, insulin, flow cytometry, real-time expression profile, cytokines and western blot were tested for normality by Kolmogorov-Smirnov test and transformed to natural logarithms or ranks as appropriate when not normally distributed. Flow cytometry data of various organs and cell phenotypes, expression profile data, anti-GAD65 antibody measurement data, insulin and glucose tolerance test data were statistically analyzed by SAS MIXED procedure (version 9.3, SAS Institute, Inc., Cary NC, USA). Analyses were done by a 2 × 2 factorial ANOVA for the factors of group and gender for the main effects of group, gender and their interaction. When a significant main effect or interaction were detected, Student’s unpaired t-tests were used to determine differences in means between two groups or between two genders. For glucose and insulin concentrations after GTT test in both treated and nontreated groups, analysis was done separately for male and female mice with a two-way ANOVA for main effects of treatment, time and their interaction. The significant main effects were further tested to locate the difference in means by least significant difference test (for difference among time points) and Student’s unpaired t-tests (for difference between two treatments). The statistical significance threshold was at p ≤ 0.05. Probabilities between p > 0.05 and p ≤ 0.10 were regarded as approaching significance. Data were presented as the mean ± SEM.

## Results

### elF5A inhibition delays onset of diabetes by altering the immune response

**Figure 1:**
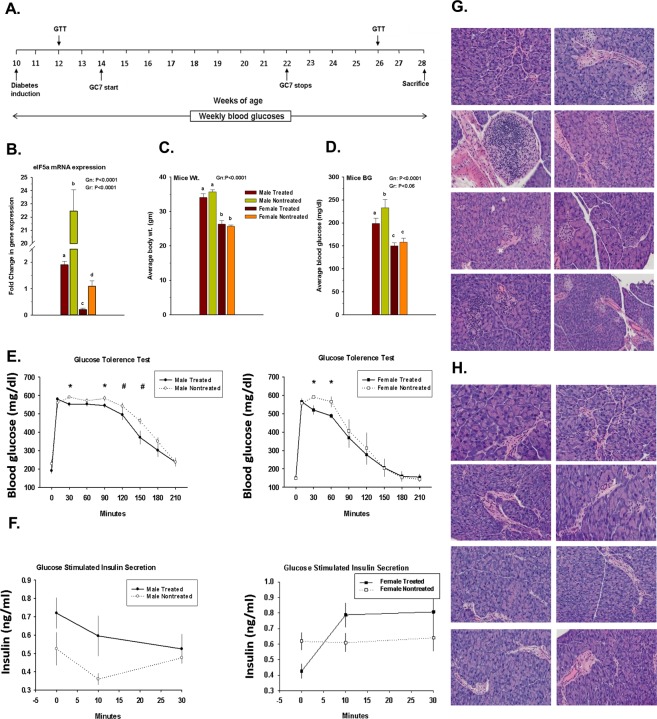
Effect of GC7 treatment: (**A**) Experimental Design Scheme, (**B**) elF5A mRNA expression, (**C**) Mouse body weight (gm) and (**D**) Fasting blood glucose (mg/dl) (bar graph) (n = 6–8 per group). Intraperitoneal administration of GC7 significantly reduced the elF5A mRNA expression in the treated groups (n = 4–6 per group). Administration of GC7 reduced the fasting blood glucose but the effect was significant only in the male treated group. Statistical significance was determined at P < 0.05. Lowercase letters (a–c) identify significant differences among the groups. (**E**) Glucose tolerance test (GTT) in male and female GC7 treated and nontreated groups at the 26^th^ week. Males had significantly (*) lower blood glucose at 30 and 90 min. and approaching significance (#) at 120 and 150 min. time points (**E**) as compared to nontreated male, whereas, females showed significant differences (*) only at 30 and 60 min. time points as compared to nontreated females. (**F**) Glucose-stimulated insulin secretion assay (GTT Insulin). Note that the increase in insulin secretion was observed in both male and female treated groups (n = 6–8 per group). (**G**,** H**) Pancreatic frozen sections were stained with hematoxylin/eosin (H&E) for histological identification and localization of lymphocytic infiltration. Treated mice pancreases have a higher degree of infiltration with partially digested islet architecture (**G**) whereas, in the nontreated group, the pancreases were small with digested islet architecture (**H**). Note: Treated group have a significantly higher CD8 count in the pancreas as compared to nontreated group irrespective of gender bias (Fig. 2). Gr indicates group; Gn indicates gender. Statistical significance was determined at P < 0.05. Lowercase letters (a–c) identify significant differences among the groups.

Previously, we have shown a gender bias in the expression of elF5A in the pancreas of our humanized mice^44^. In the present study, we again confirmed the biased elF5A expression and that the GC7 treatment significantly inhibited elF5A expression in both males and females (Fig. 1B). No significant difference was observed in the average body weight between the treated and nontreated groups throughout the experimental period (Fig. 1C). Average fasting blood glucose level was lower in GC7 treated group than in the nontreated group with a significant difference only in males (Fig. 1D). GTT at 26 weeks of age showed males had significantly (P < 0.05) lower blood glucose at 30 and 90 min. and approaching significant (P = 0.06 to P < 0.1) lower blood glucose at 120 and 150 min. time points (Fig. 1E). In contrast, females in the treated group showed lower (P < 0.05) levels only at 30 and 60 min. time points as compared to nontreated females.

Insulin release from beta cells is a biphasic response involving a first rapid phase of exocytosis of available insulin granules, and a second sustained phase of production and release in response to glucose signaling^52^. In the present study, GSIS showed a differential response based on gender. Males show higher insulin release in the first phase (0–10 min) whereas the females showed higher release in the second phase (10–30 min) (Fig. 1F). The elF5A inhibition improved the first phase of insulin release in males and the second phase of insulin release in females.

Pancreatic frozen sections were stained with hematoxylin/eosin (H&E) for histological identification and localization of islets and lymphocytic infiltrates. We observed an improved effect of GC7 treatment on islet architecture. In the treated group, islets were fully infiltrated but with less disrupted architecture (Fig. 1G); whereas in the nontreated group the islets were small with a completely disrupted architecture (Fig. 1H).

### Immune resetting in the pancreatic microenvironment following elF5A inhibition

**Figure 2:**
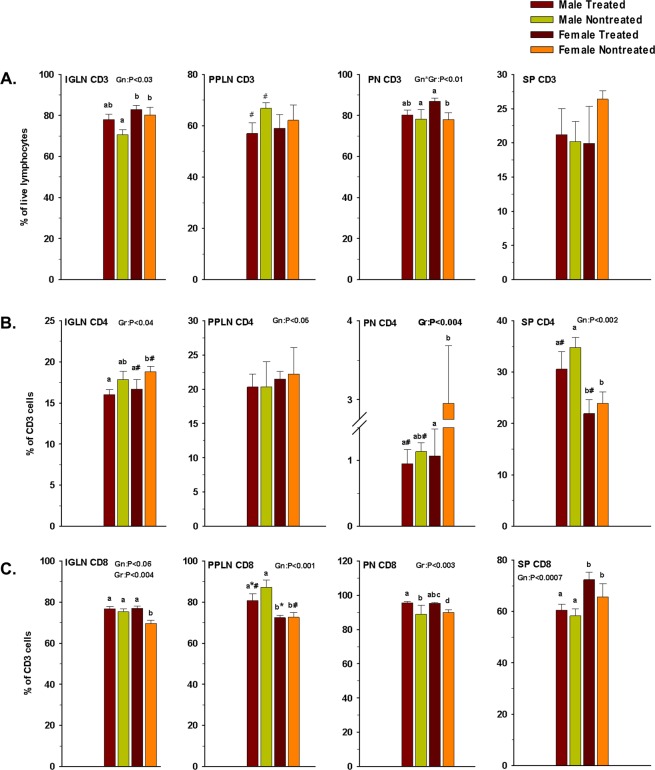
Effect of GC7 treatment on CD3, CD4 and CD8 cells. CD3 (**A**), CD4 **(B)** and CD8 (**C**) cells at inguinal lymph nodes (IGLN), pancreas (PN), peri-pancreatic lymph nodes (PPLN), and spleen (SP) in GC7 treated and nontreated male and female mice (n = 4–6 per group) are shown. CD3 and CD4 cells percentage were lower in the PN of treated mice whereas, CD8 count was significantly higher in the PN of treated mice. Gr indicates group; Gn indicates gender. Statistical significance determined at P < 0.05. Lowercase letters (a–c) identify significant differences among the groups. Means with different superscript (* or #) have an approaching difference (P = 0.06 to P < 0.1) among the groups or genders.

Immune T cell profiling of most lymphoid organs of diabetic mice [inguinal lymph nodes (IGLN), peri-pancreatic lymph nodes (PPLN) and spleen] are known to be rich in both CD4 and CD8 T cells, whereas the pancreas is mostly infiltrated by CD8 T cells (Fig. 2C). The CD8:CD4 ratios in the screened lymphoid organs ranged 4:1 to 3:1 in the IGLN, PPLN and spleen whereas there was a huge disparity in the pancreas. In mice and human pancreas’ of diabetic individuals, CD4 T cells account for only 2–3% while CD8 T cells account for 80–90% of the total CD3 positive cells. Coppieters *et al*. have reported a similar observation in humans^37^, where it has been demonstrated that “autoimmune, islet-reactive CD8 T cell species infiltrated islets from individuals with recent-onset and longstanding T1D”.

elF5A inhibition reduced the CD4 T cell population. The spleen showed no significant reduction, whereas IGLN and PPLN showed some reduction, the pancreas of the female mice showed a significant reduction, and in the pancreas of the male mice the effects were approaching significance (Fig. 2B). The effect of elF5A inhibition on CD8 T cells was minimal at IGLN and SP. Interestingly in PPLN, CD8 T cells reduced marginally, whereas in PN it increased significantly (Fig. 2C).

### elF5A inhibition enriches Treg population

**Figure 3:**
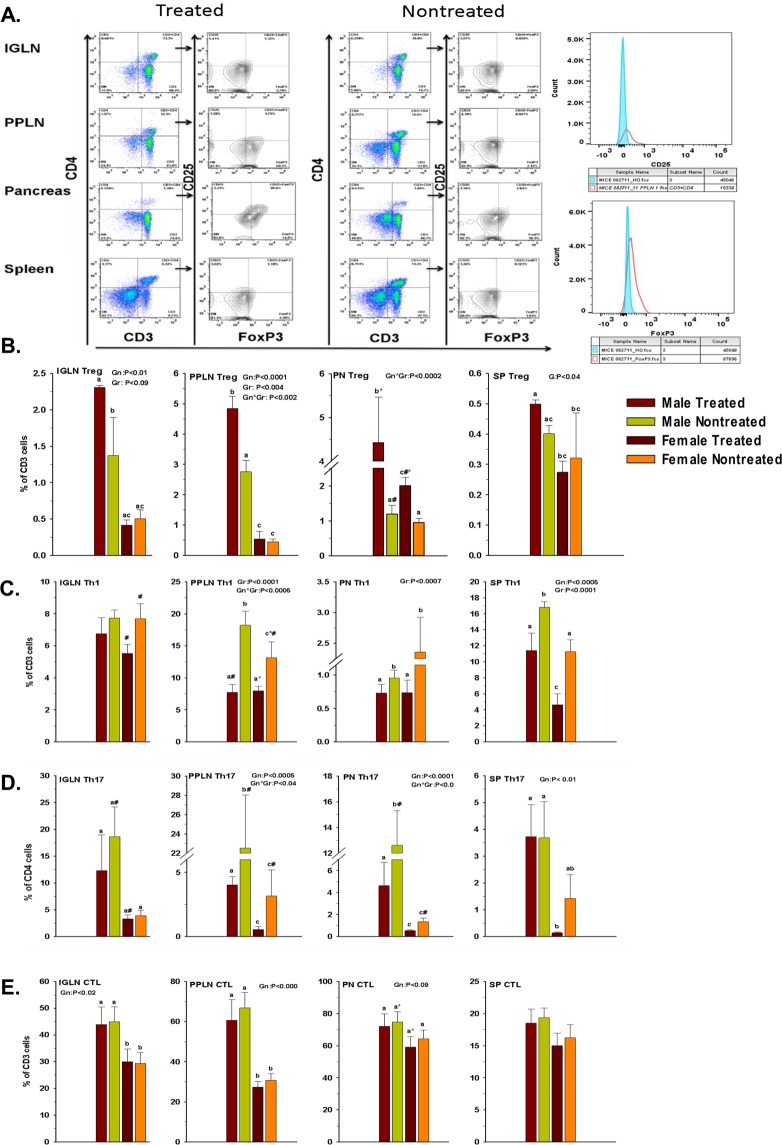
(**A**) Representative flow cytometry analysis (dot plots) gated for CD4 positive T cells and further analyzed for FOXP3 and CD25 (contour plots) of Inguinal lymph nodes (IGLN), Peri-pancreatic lymph nodes (PPLN), Pancreas (PN) and Spleen (SP) in GC7 treated and nontreated mice. (**B**) Effect of GC7 treatment on Treg cells. Statistical differences (bar graphs) of Treg population are shown. Note that the Treg cells were significantly increased in all treated groups and the difference became more significant closer to the pancreas (PPLN and PN) (n = 4–6 per group). (**C**) Effect of GC7 treatment on Th1-cell population (CD3 + CD4 + IFNγ), (bar graphs) (n = 6–8 per group). Note that the Th1 cells were significantly reduced in treated groups and the reduction becomes more significant at PPLN, PN and SP. (**D**) Effect of GC7 treatment on Th17 cells (CD3 + CD4 + IL17). Statistical differences (bar graphs) of Th17 cells in treated and nontreated mice (n = 6–8 per group) are shown. Note that Th17 cells were significantly reduced in treated groups and the difference becomes more significant closer to the pancreas (PPLN and PN). **(E)** Effect of GC7 treatment on antigen-specific cytotoxic CD8 T cells (CTLs) (CD3 + CD8 + IFNγ). Statistical differences (bar graphs) of CTLs cells (n = 6–8 per group) are shown. Note that CTLs cells were reduced in treated groups and that the difference only becomes apparent at PPLN and PN but no overall effect was observed. Gr indicates group; Gn indicates gender. Statistical significance was determined at P < 0.05. Lowercase letters (a–c) identify significant differences among the groups. Means with different superscript (* or #) have an approaching to significance difference (P = 0.06 to P < 0.1) among groups or genders.

Previously, we have demonstrated that elF5A inhibition improves the Treg population in the pancreas’ of type 1 diabetic mice^44^. Here, we analyzed in more detail the Treg response in and around the pancreas after elF5A inhibition (Fig. 3A, B). We observed that the enrichment of Tregs was not only limited to the pancreas but are also significantly present in the IGLN, PPLN and spleen (Fig. 3A, B). In males, elF5A inhibition enriched Tregs in all lymphoid organs and the enrichment was more significant closer to the pancreas, whereas in females the significant enrichment (p < 0.05) of Treg population was observed only in pancreas following elF5A inhibition.

### elF5A inhibition reduces Th1 and Th17 cells

The inflammatory nature of TID suggests that Th1 and Th17 cells should be elevated; we thus measured the effect of elF5A inhibition on IFNγ producing CD4 T cells (Th1) and IL17 producing CD4 T cells (Th17). In the nontreated group, we observed the infiltration of both cell types in the pancreas and elevated numbers in IGLN, PPLN and spleen. In the treated group, inhibition of elF5A significantly reduced the frequency of Th1 cells at all lymphoid organ sites, especially closer to the pancreas (Fig. 3C). The reduction in Th17 cell numbers was highly significant, approaching 60%, in PPLN and pancreas but was not-significant in IGLN and spleen (Fig. 3D) in comparison to the nontreated group. Interestingly, the reduction was more pronounced in males as compared to females, in accordance with their elF5A expression (Fig. 1B). This observation also implies that most of the islet-specific Th17 cell recruitment may have occurred at PPLN and pancreas level (Fig. 1G).

### elF5A inhibition has least effect on CTLs

CTLs are the main effector cells executing the destruction of β-cells. We show here that in our diabetic mice, IFNγ producing CD8 T cells are the main immune subset present in the pancreas and the sentinel lymph nodes (PPLN, IGLN) (Fig. 3E). This observation supports that T1D in our mouse model mimics T1D of humans^3–5^. Following the elF5A inhibition, we did not observe any significant reduction in the CTL population in all of the lymphoid organs screened.

### Differential expression of elF5A, hypusinated elF5A and DHPS in CD4 and CD8 T cells

**Figure 4:**
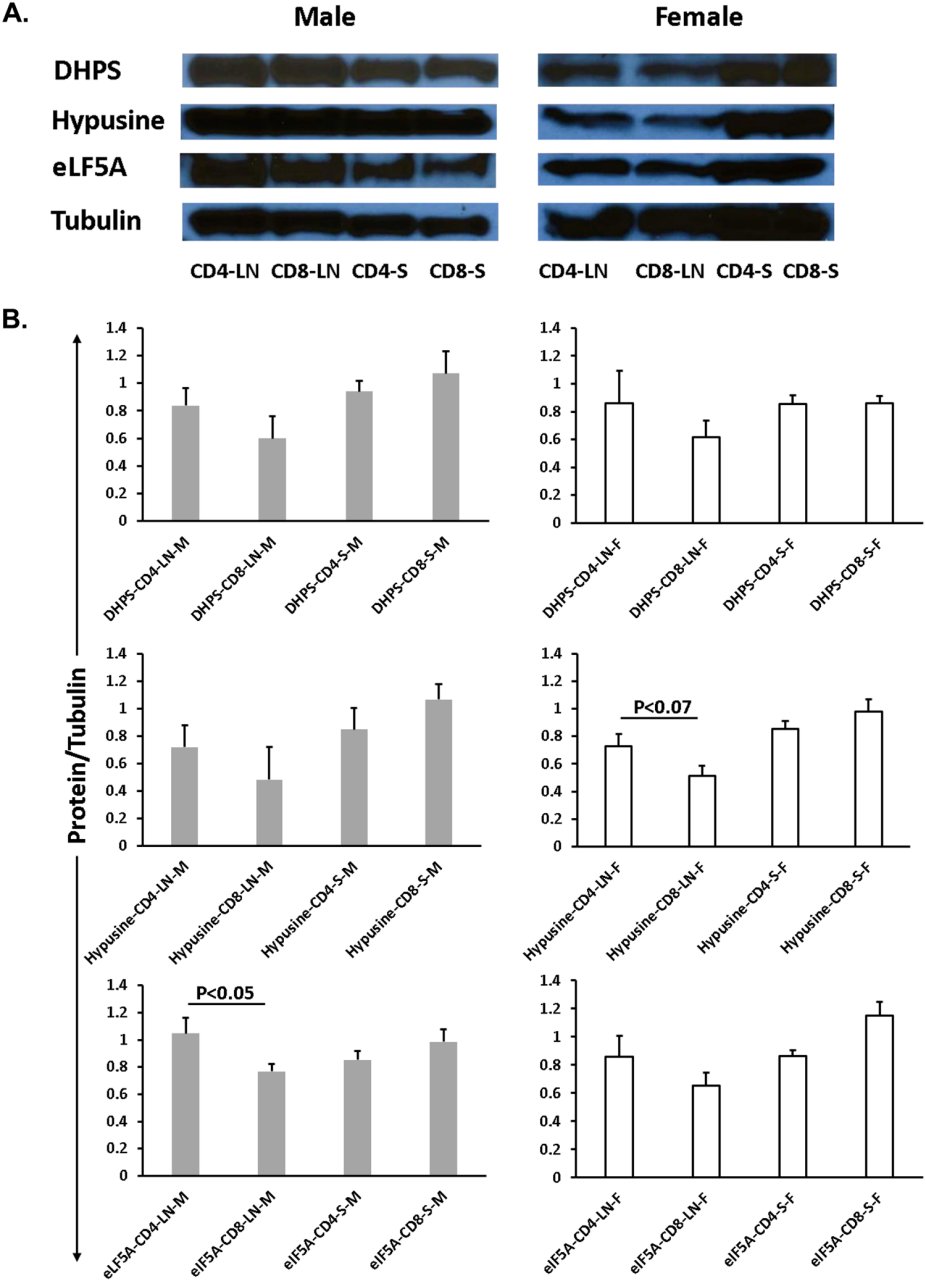
Differential expression of elF5A, Hypusinated elF5A and DHPS in CD4 and CD8 T cells. **(A)** Western blot of CD4 and CD8 lymphocytes isolated from pancreatic lymph nodes (LN) and spleen (S). All the bands present in male/female samples were extracted from the respective single blot with the same exposure time. **(B)** DHPS, Hypusine, and elF5A were less expressed in CD8 T cells of pancreatic lymph nodes whereas the expression of these proteins were higher at splenic CD8. Note: elF5A protein expression was significant (P < 0.05) higher in the CD4 cell lysate of male LN.

CD4 and CD8 lymphocytes were isolated from peri-pancreatic lymph nodes (LN) and the spleen (S). The protein blot of DHPS, hypusinated elF5A and elF5A proteins were compared and results revealed that DHPS, hypusinated elF5A and elF5A were expressed in higher concentrations in CD4 single cell lysates as compared to CD8 single cell lysates from LN. However, we noticed the opposite results in splenic single cell lysates of CD4 and CD8 cells (Fig. 4A, B). Interestingly, we also observed a significantly higher expression (P < 0.05) of elF5A protein in the CD4 cell lysate as compared to CD8 cell lysate of male LN (Fig. 4B). Higher expression of elF5A in CD4 as compared to CD8 T cells has been reported in BioGPS (http://biogps.org/#goto=genereport&id=1984).

### elF5A inhibition changes the tissue-specific T cell ratios

**Figure 5:**
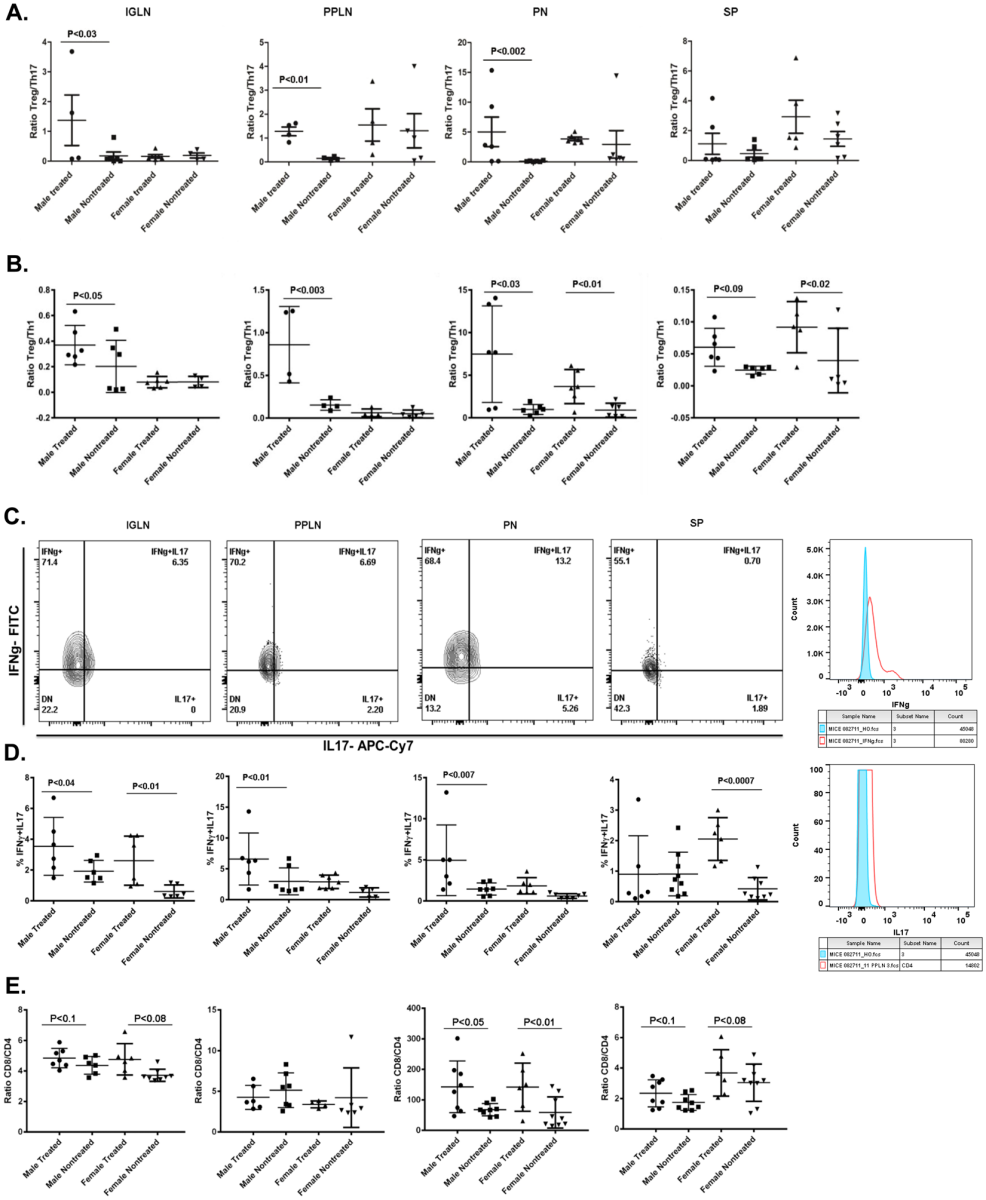
(**A**) Effect of GC7 treatment on Treg/Th17 ratio. Treg/Th17 ratios at inguinal lymph nodes (IGLN), Pancreas (PN), Peri-pancreatic lymph nodes (PPLN), and Spleen (SP) in GC7 treated and nontreated male and female mice (n = 4–6 per group) are shown. Note that the difference in Treg/Th17 ratios became significant closer to the pancreas (PPLN and PN). (**B**) Effect of GC7 treatment on Treg/Th1 ratio. Treg/Th1 ratios at IGLN, PN, PPLN, and SP in GC7 treated and nontreated male and female mice (n = 4–6 per group) are shown. Treg/Th1 ratio was higher in the IGLN, PPLN, PN and SP of male treated mice, whereas in female Treg/Th1 ratio was higher at PN and SP. (**C**) Effect of GC7 treatment on Th1 and Th17 cells plasticity. Upper panel represents a contour plot of CD4 + T cells co-expressing IL-17 and IFNγ at IGLN, PPLN, PN and SP. In males, the IFNγ + IL17 + CD4 cells were significantly increased in IGLN, PPLN and PN whereas in females the significant increase was observed only in SP and IGLN. (**D**) Effect of GC7 treatment on CD8/CD4 ratios at IGLN, PN, PPLN, and SP; CD8/CD4 ratios were increased in the pancreas, IGLN and spleen whereas the ratio decreased in PPLN of both males and females. Note: This decrease in ratio implies that eIF5A inhibition reduced the CD8 T cell count in the PPLN only, site of recruitment of CD8 T cells in T1D (n = 4–6 per group).

The outcome of autoimmunity usually depends on the balance between the pro-inflammatory and anti-inflammatory signals at the specific tissue sites. Thus, we determined the effect of eIF5A inhibition on the ratios of effector T cell populations in different lymphoid organs of the treated group. The analysis revealed that the Treg/Th17 ratios were increased significantly in the pancreas, IGLN and PPLN of males, whereas the effect on the ratio in these tissues was non-significant in females (Fig. 5A). No difference was observed in Treg/Th17 ratio in the spleen of either male or female mice. The ratios of Treg/Th1 in males were significantly elevated in pancreas, IGLN and PPLN, but were trending significantly higher in the spleen (Fig. 5B). In females, the Treg/Th1 ratios showed significant differences only in the pancreas and spleen but no significant differences were observed in IGLN and PPLN.

Th17 cells are plastic and have been described to acquire Th1 phenotypes. Thus, we evaluated the effect of elF5A inhibition on the population of CD4^+^ T cells co-expressing IL17 and IFNγ, a transitionary cell type between Th17 and Th1 cells. In males, the IFNγ + IL17 + CD4 cells were significantly increased in IGLN, PPLN and pancreas following eIF5A inhibition, whereas in females the significant increase was observed in the spleen and IGLN only (Fig. 5C). This observation suggests that the eIF5A inhibition may influence the plasticity of T helper cells.

We further compared the CD8/CD4 ratio in different lymphoid organs. The analysis revealed that the ratios were increased in the pancreas, IGLN, and spleen following elF5A inhibition, whereas decreased in PPLN of both males and females (Fig. 5D). This decrease in ratio implies that the eIF5A inhibition reduced the CD8 T cell count (Fig. 2C) or increased the CD4 T cell subsets (Tregs, Fig. 3B) in the PPLN (site of activation and proliferation). Overall this data emphasizes the impact on the immunological outcome of eIF5A inhibition which seems to occur both at the sites of activation and execution.

### Effect of elF5A inhibition on serology

**Figure 6:**
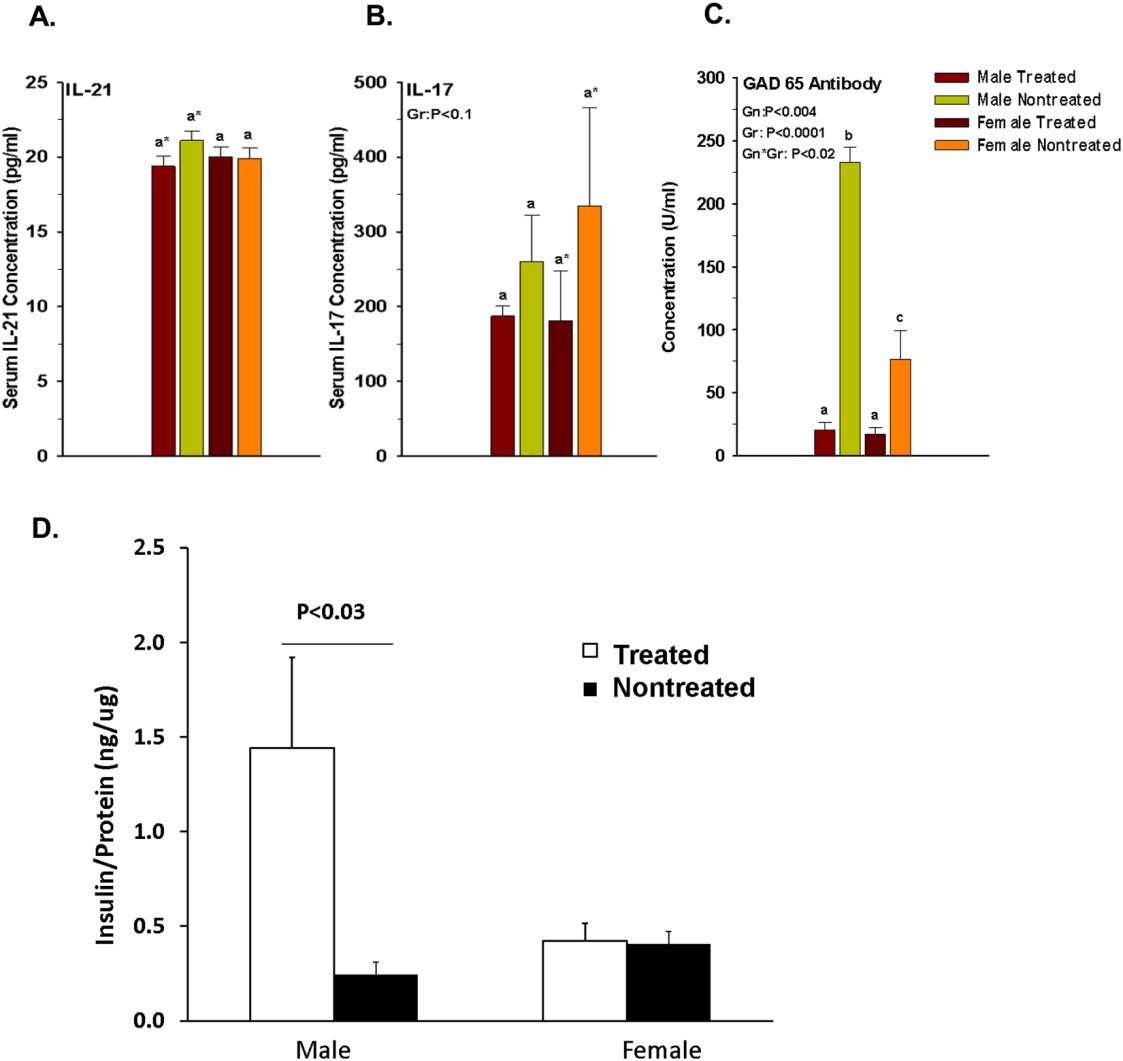
(**A**) Effect of GC7 treatment on Mice serum concentration of IL-21 (**B**) IL-17 in GC7 treated male and female mice. Note that the IL-17 concentration directly correlates with the IL-21 serum concentration. Note that serum IL21 concentration was reduced to approaching significance in treated male and serum IL17 concentration was reduced to approaching significance in the treated female group. (**C**) Effect of GC7 treatment on anti-GAD65 antibody production. Administration of GC7 significantly reduced the GAD65 antibody production in the treated group (n = 4–6 per group). Note that elF5A expression directly correlates with the severity of T1D and GAD65 antibody production. (**D**) Effect of GC7 treatment on pancreatic beta cell functionality. Administration of GC7 significantly increased the functionality of beta cells (n = 4–6 per group). The total insulin production was significantly increased in the male treated group. Gr indicates group; Gn indicates gender. Statistical significance was determined at P < 0.05. Lowercase letters (a–c) identify significant differences among the groups. Means with different superscript (* or #) have an approaching to significance difference (P = 0.06 to P < 0.1) among the groups.

Chronicity of T1D correlates with IL17 and IL21 concentrations^53^; therefore, we measured the serum cytokine levels in our mice at the end of the experiment. The elF5A inhibition reduced serum IL17 and IL21, but the effect was not significant (Fig. 6A, B).

We also measured anti-GAD65 antibodies in the sera of mice. Serum anti-GAD65 antibodies were detected in both groups (treated/nontreated) and their titers correlated with the serum glucose levels and GTT. Inhibition of elF5A significantly reduced the antibody titer both in males and females (Fig. 6C) and this reduction correlated with the delay of diabetes onset/progression.

### elF5A inhibition improves the total pancreatic insulin content

We have shown a gender bias in the pancreatic elF5A expression in our humanized mice (Fig. 1B). A similar kind of gender bias was also seen in the total insulin content of the pancreas and the elF5A inhibition significantly increased the pancreatic insulin content in the male treated group only (Fig. 6D).

### elF5A inhibition reduces the ER stress gene profile in the pancreas

**Figure 7:**
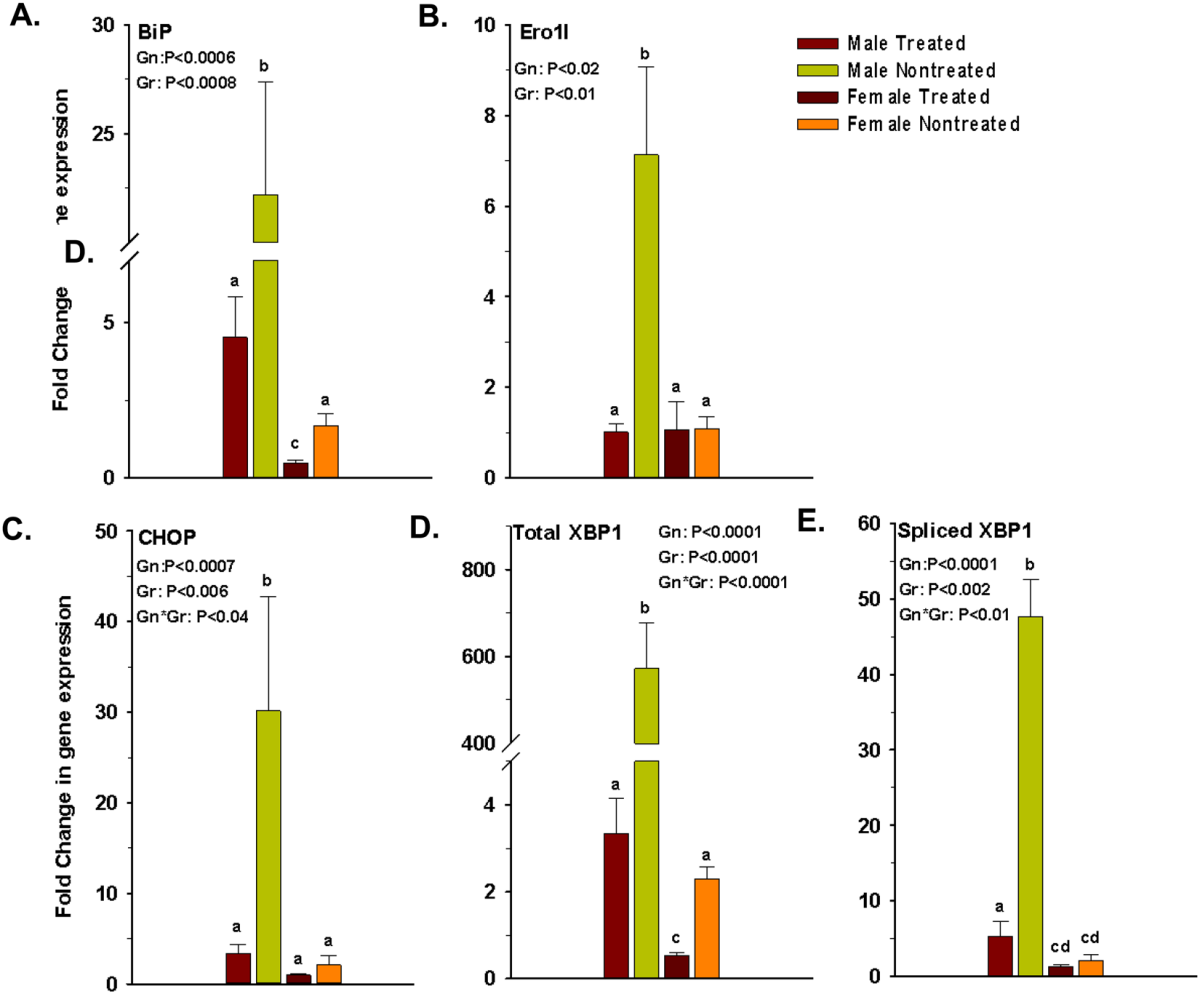
Effect of GC7 treatment on markers of ER stress in islet microenvironment. Administration of GC7 significantly reduced the expression of ER stress genes (**A**) *BiP*, (**B**) *Ero1l*, (**C**) *CHOP*, (**D**) total *XBP-1* and (**E**) spliced *XBP-1*s in the pancreatic islet microenvironment of treated mice (n = 4–6 per group). Gr: group; Gn: gender. Statistical significance determined at P < 0.05. Lowercase letters (a–c) identify significant differences among the groups.

A pro-inflammatory bias in the pancreatic microenvironment induces ER stress in β-cells. We, therefore, studied the effect of eIF5A inhibition on the expression of ER stress-related genes in the pancreas. Our analysis revealed that elF5A inhibition in the treated group significantly reduced the expression of BiP, Ero1l, CHOP, total XBP-1 and spliced XBP-1s in both genders compared to control group, but the effects were more pronounced in males. The total and spliced XBP 1 which were highly expressed in nontreated group, showed a huge decrease after eIF5A inhibition in both genders (Fig. 7D, E). Expression of BiP, CHOP and Ero1l was also high in nontreated diabetic mice but the expression was reduced significantly following the eIF5A inhibition (Fig. 7A–C).

## Discussion

In this study, we were able to delineate the role of autoreactive T helper cells and CTLs in the mediation of T1D as a result of a specific intervention. The eIF5A inhibition reduced the pro-inflammatory bias in the pancreatic microenvironment by reducing Th1 and Th17 cells, and enriching Tregs. This enrichment helped to improve the functionality of β-cells in terms of insulin release and reducing ER stress; however, it did not abrogate the CTL mediated destruction of β-cells. Overall, our study illustrates that interventions targeting only the T helper cell subsets may not be able to revert T1D (at least in our humanized mouse model) until the interventions to restrain autoreactive CTLs in the islet microenvironment are combined. This may also hold true for interventions for human T1D.

eIF5A is a small, highly conserved protein necessary for translational elongation of specific mRNA transcripts, especially of proteins involved in G1 to S cell cycle transition^54^ and cytotoxic stress response^55^. Few studies have demonstrated eIF5A’s immunological role^56,57^. Others have demonstrated that hypusinated eIF5A is essential for the expression of CD83 in DCs correlating to their maturation^58^. In the context of T1D, eIF5A participates in the inflammatory cascade leading to β cell dysfunction during the development of diabetes in NOD mice^43^. Most importantly, Colvin *et al*. previously demonstrated that eIF5A inhibition by GC7 administration in NOD mice causes selective reduction of diabetogenic Th1 cells in the pancreatic lymph nodes^45^.

This current study and our previous study^44^ demonstrated that eIF5A inhibition by GC7 has a more profound effect on T helper cell compartments (Th1, Th17 and Tregs) as compared to the CTL compartment in our humanized mouse model of T1D.

The novelty of our findings can be enumerated as follows: (1) We revealed that the plasticity of T helper cells seems to be ideal in providing the pro-inflammatory bias for induction of T1D; and that plasticity gives room to manage T1D in the early stage of the disease. (2) We demonstrated that inhibition of elF5A helps enrich Treg/Th17 ratios and is a positive development in delaying the onset of T1D. (3) We proved that reduction in pro-inflammatory cytokines reduces ER stress in secretory endocrine cells of the pancreas, which in turn sustains insulin production. (4) We provided evidence that by reducing the pro-inflammatory cytokine milieu late in the disease, T1D cannot be reverted unless antigen-specific CTLs are halted.

The differential gender bias of elF5A expression may explain the overall lower response in female mice to the elF5A inhibition. This bias was also seen in the total insulin content of the pancreas (Fig. 6D). The elF5A inhibition showed a significant increase in the pancreatic insulin in males only. The gender bias in incidence and aggressiveness of T1D is also observed in humans where global male: female incidence ratio is 1.47^59^, in contrast with that observed on NOD mice^60^. Differential expression of elF5A in CD4 and CD8 T cells has been reported in BioGPS and similarly demonstrated in our western blot results (Fig. 4B).

Inhibition of elF5A reduces the pro-inflammatory bias by reducing the number of antigen-specific Th1 cells in the target lymphoid organs. In our mice, the effect of inhibition was more pronounced closer to the pancreas. The reduction in Th1 cells may be due to a change in the phenotype of T helper cells to Tregs, or may be due to subsequent events that create other T-helper cell subsets^61^. It has been also reported that Treg cells can be converted into IL17 expressing CD4 cells in a proinflammatory cytokine milieu^38^. Specific transcription factors have been identified which orchestrate the differentiation program of T helper subsets; mainly TGF-β and IL6 are important for Th17 differentiation; whereas TGF-β, retinoic acid and IL-2 dominance is prerequisite for iTreg cells differentiation^62^. Th1 cells play an important role in priming CTLs as it has been demonstrated that Th1 cells could acquire major histocompatibility complex/peptide I complexes and costimulatory molecules by dendritic cell activation, and further stimulate naïve CD8^+^ T cell proliferation and activation of CTLs^63^. Increasing levels of Th1 was shown to correlate with the progression of diabetes in NOD mice^64^. Inhibition of elF5A in the treated group reduced the Th1 cells in and around the pancreas (PPLN and PN) in our humanized mouse model. The inhibition of elF5A significantly reduced the Th1 response at all sites but the effects were more significant closer to the pancreas.

In our humanized mouse model, upon GAD65 immune response induction, Th17 cells become abundantly present in the pancreatic microenvironment as seen in the nontreated group. Inhibition of elF5A in the treated group significantly reduced the population of Th17 cells and the reduction in Th17 cells likely helped Treg cells proliferate. Alternatively, inhibition of elF5A may have induced plasticity of Th17 cells towards Treg cells^61^. The effect of elF5A inhibition on the reduction of Th17 cells was more pronounced at the IGLN, PPLN and PN of male as compared to female mice. Whereas inhibition of elF5A enriched the Treg cells in the pancreatic microenvironment, the enrichment was significantly higher in lymphoid organs of both males and females in the treated group. Our findings suggest that Treg/Th17 ratio determines the fate of beta cells in our mouse model. Here we further showed that inhibition of elF5A reduced the Th17 cell population and improved the Treg population in the pancreatic microenvironment. The increment in the Treg/Th17 ratio after elF5A inhibition directly correlated with the beta cell functionality and insulin production.

Differentiation of CD4 T cells to different subsets of T cells are cytokines milieu dependent. Here we have modulated the cytokines milieu by inhibiting elF5A which may have had a role in inducing plasticity of different T helper subsets towards Tregs. However, our findings raise the question of whether a reduction of inflammatory cells can abrogate ongoing T1D due to auto-antigen specific CTLs. We have dissected the role of immune cells in the pathogenesis of the disease; by inhibiting elF5A, we were able to reduce the pro-inflammatory bias. The overall effect was that elF5A inhibition improved the functionality of beta cells in terms of GTT and total pancreatic insulin content. However, reducing the pro-inflammatory milieu was unable to change the fate of T1D outcome. Our findings suggest that autoreactive CTLs should also be a target of immune based treatment of T1D.

CD8/CD4 T-cell ratio represents an index of immunological balance between T-cytotoxic cells and T-helper^65,66^. In the case of children with diabetes, positive for complement fixing islet cell antibodies (CF-ICA), there was a consistent increase in the CD8/CD4 lymphocyte ratio before the clinical onset of the T1D^66^. Therefore, increments in the CD8/CD4 ratio appear to be a marker of T1D progression. In our study, we observed that elF5A inhibition had an opposite effect on all the lymphoid organs like IGLN, PN and Spleen as CD4 count were reduced in these three organs (Figs 2 and 4D), whereas elF5A inhibition induced a reduction in the CD8/CD4 ratio at PPLN. This reduction is important because this is the site of CD8/CTLs proliferation and licensing as evident by the histochemistry of the pancreas in the treated group (Fig. 1G).

IL21 is mainly secreted by Th17 cells in autoimmune diseases, and has a major role in driving terminal B-cell differentiation to plasma cells while having a major stimulatory effect on antigen-specific autoreactive T-cells. It has been shown that IL21 production correlates with increased numbers of Th17 cells and decreased numbers of Treg cells in patients with SLE^67^. It has also been reported that Th17 cells could be involved in the control of the local and systemic inflammatory responses in chronic emphysema by upregulating the CD8^+^ cytotoxic T-cell function^68^. Conversely, we show that inhibition of elF5A marginally reduces the CTLs pool at PPLN in our T1D model (Fig. 3E). This effect may be because of reduction in serum IL21 and IL17 cytokines, which is important for the activation and maintenance of CTLs^68–71^. Along with the promotion of CD8 cytotoxic responses, it has been shown that in emphysematous mice, Th17 and IL21 cells are positively correlated with their lesions^68^. Here we want to state that inhibition of elF5A reduces the IL21 expression in male (Fig. 6A) and IL17 expression in both the genders (Fig. 6B), which may be due to a change in pro-inflammatory bias, and may help in marginal reduction of CTLs in peri-pancreatic lymph nodes of mice (Fig. 3E).

Pancreatic β-cell endoplasmic reticulum (ER) shows an adaptive response even in normal physiological conditions. Both acute (1–3 hours) and chronic (≥24 hours) exposure to high glucose (≥16.7 mM) have been shown to induce ER stress in mice^72^. However, in the case of T1D, the ER stress level is exaggerated by the immune cells infiltrating the pancreatic islets, secreting various pro-inflammatory cytokines, which directly affect the islet microenvironment and exacerbate ER stress. When studying the effect of cytokines on ER stress response, isolation of individual islets is not suitable because the process itself exacerbates ER stress^73^. To manage inflammation, metabolic, and other stressful adverse conditions, the organelle has evolved an adaptive response. Here, the effect of inflammation on ER stress was studied by measuring the expression of the ER stress genes in the whole pancreas (Fig. 7). When the ER is loaded with an excess of protein folding work, the UPR signaling pathways will be activated. In the first line of defense, stressed cells try to survive through the arrest of transient translation of nascent proteins. Secondly, the export of misfolded proteins for degradation known as ER-associated degradation (ERAD). In the third stage, ER increases the folding capacity of the affected ER by induction of chaperones and folding enzymes. Prolonged ER stress impairs homeostasis and induces apoptosis^74,75^. X box-binding protein (XBP)1 overproduction is part of the ER stress response and overexpression increases the aggressiveness of the autoimmune diseases^76,77^. Similar patterns have been observed in our mouse model (Fig. 6C, D). Basal expression of elF5A is essential for ER-to-Golgi vesicle transport^78,79^, whereas DHS promotes the differentiation and proliferation of T Helper Type 1 (Th1) Cells in T1D^45^. Inhibition of elF5A using DHS inhibitor “GC7” significantly reduced the XBP-1 and spliced XBP-1s expression in the pancreas of our mouse model (Fig. 7D, E). Spliced XBP-1 helps the ER in compensating mild to moderate ER stress^80^. A correlation of *XBP-1* gene expression level with antibody production has also been shown^80^. The expression of XBP-1 protein is required for the transcription of a subset of class II major histocompatibility genes^77^. XBP-1, in turn, controls the expression of IL6 which promotes plasma cell growth and production of immunoglobulins^81^. Our results show that XBP-1 gene expression is correlated with the anti-GAD65 antibody production, which was reduced significantly with the inhibition of elF5A (Fig. 6C, D). BiPs or HSPA5 is a 78 kDa ER chaperone protein, serving as an ER stress sensor. Under oxidative and functional stress, BiP overexpressed and compensates ER stress (adaptive phase). According to the results, elF5A inhibition significantly reduced BiP in both male and female mice in the treated group and reduced the ER stress level in the pancreas (Fig. 7A). Prolonged ER stress impairs homeostasis to compensate for the workload of the UPR. Endoplasmic reticulum overexpresses CHOP, a transcription factor belonging to the bZIP family (alarm/apoptosis phase). Upon activation, CHOP suppresses anti-apoptotic protein BCL-2, which may induce beta cell apoptosis^82^. Here we have shown that inhibition of elF5A significantly reduces CHOP expression in both male and female mice in the treated group, but the effect was more significant in males (Fig. 7C). Therefore, inhibition of elF5A may protect the beta cells from ER stress mediated apoptosis, as evidenced by immunohistochemistry of treated mice pancreas (Fig. 1G). As mentioned, the pancreatic islet microenvironment of our T1D mouse model was infiltrated with Th1, Th17 and CTLs cells, which lead to high concentrations of pro-inflammatory cytokines and IL17, which likely acerbated generation of ER stress in islet/beta cells. This may have lead to secretion of reactive oxygen species, which is involved in directly inducing ER stress to adjacent islets. We show here that *elF5a* gene expression was proportional to ER stress gene expression, anti-GAD 65 antibody production, and inversely proportional to the Treg/Th17 ratio. Inhibition of elF5A in the treated group significantly reduced the ER stress gene expression as well as the *elF5a* expression in the pancreas of mice.

## Conclusions

Altogether, our results show for the first time, that downregulation of eIF5A through inhibition of DHS favors the reversal of the Th1 mediated described processes but minimally affects CTLs. Inhibition of elF5A increases the Treg/Th17 ratio, reduces anti-GAD65 antibody production and islet/β-cell ER stress that leads to improvement in the endocrine pancreas functionality in our humanized model of T1D. Diabetes onset is delayed but progression is inevitable.
